# Deacetylated microbial biofilm exopolysaccharides: It pays to be positive

**DOI:** 10.1371/journal.ppat.1007411

**Published:** 2018-12-27

**Authors:** Hanna Ostapska, P. Lynne Howell, Donald C. Sheppard

**Affiliations:** 1 Department of Microbiology and Immunology, McGill University, Montreal, Quebec, Canada; 2 Infectious Diseases in Global Health Program, McGill University Health Centre, Montreal, Quebec, Canada; 3 Program in Molecular Medicine, The Hospital for Sick Children, Toronto, Ontario, Canada; 4 Department of Biochemistry, University of Toronto, Toronto, Ontario, Canada; 5 Department of Medicine, McGill University, Montreal, Quebec, Canada; Geisel School of Medicine at Dartmouth, UNITED STATES

## Introduction

**Fig 1:**
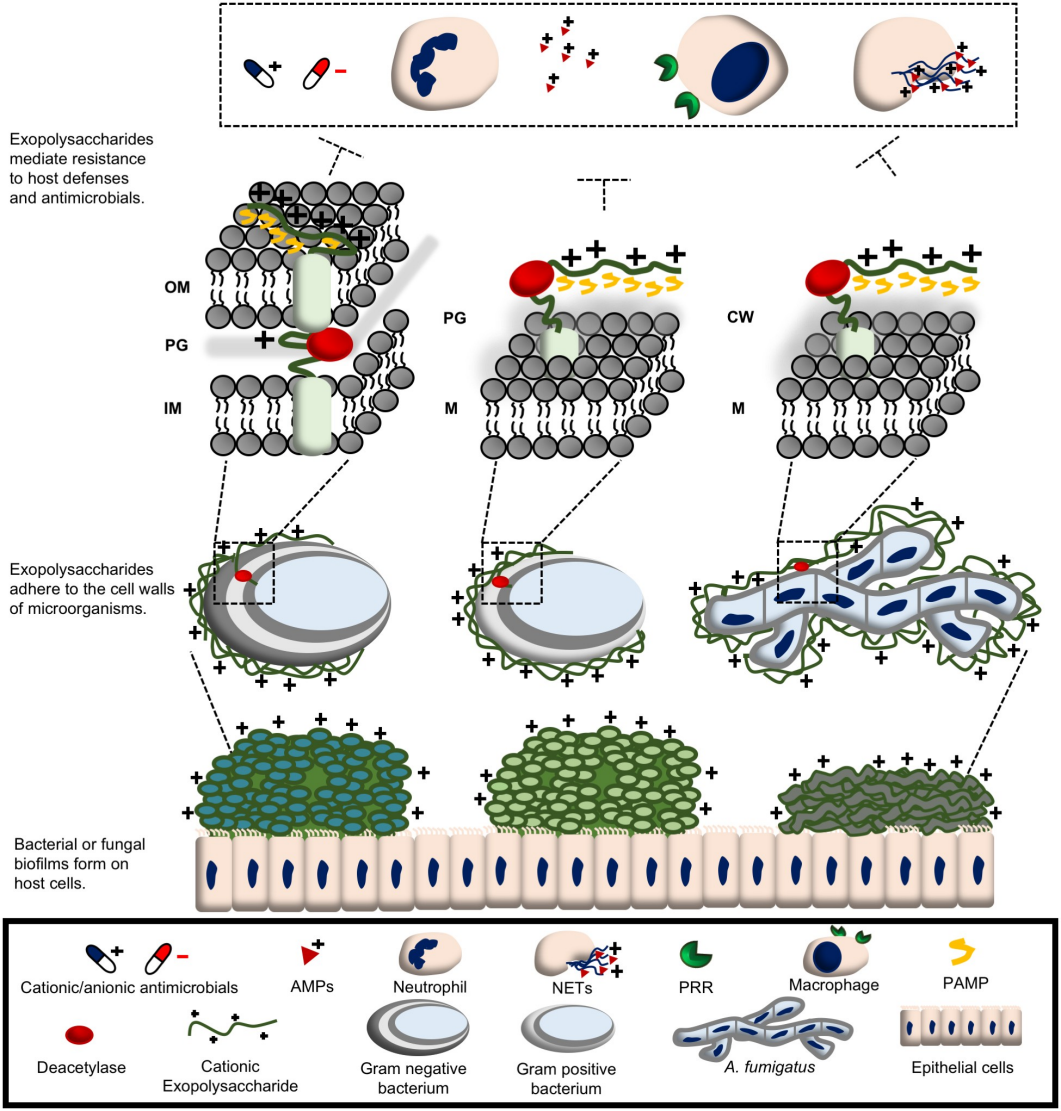
Cationic exopolysaccharides play a role in microbial pathogenesis. Deacetylated cationic exopolysaccharides mediate biofilm formation and adhere to microbial surfaces to protect microorganisms from host defenses and antimicrobials as detailed in the text. AMP, antimicrobial peptide; CW, cell wall; IM, inner membrane; M, membrane; NET, neutrophil extracellular trap; OM, outer membrane; PAMP, pathogen-associated molecular pattern; PG, peptidoglycan; PRR, pattern recognition receptor.

The production of biofilms is a common strategy used by many microorganisms during infection. Exopolysaccharides are a major component of the extracellular biofilm matrix serving to anchor organisms to surfaces, forming the structural scaffold of the biofilm, and protecting organisms from damage by hostile factors such as antibiotics and host immune defenses (Fig 1). Biochemical and genetic studies of biofilm exopolysaccharide synthesis have revealed that production of *N*-acetyl hexosamine-containing exopolysaccharides is one strategy used by diverse pathogens to facilitate biofilm formation and virulence. Following polymerization and extracellular extrusion by membrane embedded glycosyl transferases ([HexNAc]_n_ + nucleotide-HexNac → [HexNAc]_n+1_ + nucleotide), these glycans then undergo postsynthetic enzymatic deacetylation ([HexNAc)_n_ → HexN-[HexNAc]_n-1_ + acetyl group) to render them cationic. Deacetylation is critical for the function of these glycans in biofilm formation and host–pathogen interactions. This Pearl explores the role of these deacetylated cationic exopolysaccharides within the biofilm matrix in microbial pathogenesis and resistance to antimicrobial agents, and their potential as antibiofilm therapeutic targets.

## Partially deacetylated, cationic hexosamine polymers are common in biofilm forming microorganisms

A wide range of medically important microbial species produce and secrete hexosamine-rich exopolysaccharides into their self-produced extracellular biofilm matrices (Table 1). The best studied example of these glycans is poly-*β*-1,6-*N*-acetylglucosamine (PNAG), a homopolymer of *N*-acetylglucosamine (GlcNAc) residues produced by a wide range of gram-positive and gram-negative pathogenic bacteria, including *Staphylococcus* spp., *Yersinia pestis*, *Bordetella* spp., and *Escherichia coli* [1–4]. The gram-negative opportunistic pathogen *Pseudomonas aeruginosa* produces several biofilm-associated exopolysaccharides, including the linear heteropolymer Pel, composed of GlcNAc and *N*-acetyl galactosamine (GalNAc), whereas the gram-positive organism *Listeria monocytogenes* produces a *β*-1,4-linked *N*-acetylmannosamine polysaccharide decorated with terminal *α*-1,6-linked galactose (Gal) residues [5,6]. More recently, biofilm formation by the opportunistic filamentous fungal pathogen *Aspergillus fumigatus* was found to be dependent on galactosaminogalactan (GAG), a heteropolymer composed of *α*-1,4-linked GalNAc and Gal residues [7].

**Table 1:** Functions of hexosamine-rich biofilm exopolysaccharides of medically important organisms.

Organism	Polysaccharide	Deacetylase	Phenotype of deacetylase deletion strain or functional mutant strain
*Staphylococcus epidermidis*	PNAG^a^	IcaB	Devoid of cell-wall–associated PNAG [1] Impaired biofilm formation [1] Phagocytosed by neutrophils [1] Susceptible to antimicrobials [9] Susceptible to AMPs [1] Attenuated virulence in animal model [1]
*S*. *aureus*	PNAG^a^	IcaB	Devoid of cell-wall–associated PNAG [10]
*Escherichia coli*	PNAG^a^	PgaB_*Ec*_	Impaired polymer export [11] Attenuated virulence in animal model [3]
*Yersinia pestis*	PNAG^a^	PgaB_*Yp*_^b^	Devoid of cell-wall–associated PNAG [12] Impaired biofilm formation [12]
*Bordetella bronchiseptica*	PNAG^a^	PgaB_*Bb*_^c^	Forms immature biofilm [2]
*B*. *pertussis*	PNAG^a^	PgaB_*Bb*_^c^	N/A^d^
*Klebsiella pneumoniae*	PNAG^a^	PgaB_*Kp*_	N/A^d^
*Aspergillus fumigatus*	GAG	Agd3	Devoid of cell-wall–associated GAG [8] Impaired biofilm formation [8] Detection by PRR [8] NET-induced damage [13] Increased susceptibility to antimicrobials [14] Attenuated virulence in animal model [7]
*Pseudomonas aeruginosa*	Pel	PelA	Devoid of cell-wall–associated Pel [15] Impaired biofilm formation [15] Increased susceptibility to antimicrobials [16]
*Listeria monocytogenes*	EPS	PssB	Devoid of cell-wall–associated EPS [6]

^a^Previously called polysaccharide intercellular adhesion.

^b^Previously called HmsF.

^c^Previously called BpsB.

^d^Function of deacetylase remains to be studied.

**Abbreviations**: AMP, antimicrobial peptide; *Bb*, *Bordetella bronchiseptica*; *Ec*, *Escherichia coli*; EPS, exopolysaccharide; GAG, galactosaminogalactan; *Kp*, *Klebsiella pneumoniae*; N/A, not applicable; NET, neutrophil extracellular trap; PNAG, poly- *β*-1,6-*N*-acetylglucosamine; PRR, pattern recognition receptor; *Yp*, *Yersinia pestis*.

Biochemical and genetic studies have shown that, for many of these polymers, their hexosamine residues are partially deacetylated after synthesis by polysaccharide deacetylases [8]. Deacetylation of these exopolysaccharides results in a change in their physical properties. In relatively acidic environments, the exposed primary amino groups are protonated, thereby rendering the polysaccharide polycationic [1]. The polycationic nature of these exopolysaccharides is critical for the function of these glycans in microbial pathogenesis as detailed below.

## Deacetylation of hexosamine-containing exopolysaccharides underlies their adhesive properties

Deacetylation plays an important role in the synthesis, transport, and localization of many of these exopolysaccharides. Mutants of *E*. *coli*, *L*. *monocytogenes*, *P*. *aeruginosa*, *Y*. *pestis*, *Staphylococcus* spp., *Bordetella bronchiseptica*, and *A*. *fumigatus* deficient in their respective exopolysaccharide deacetylase were found to lack detectable cell-wall–associated polysaccharide [6,8,10–12,15]. In the case of *L*. *monocytogenes* and *P*. *aeruginosa*, deacetylation of their polymers seems to be required for polymer synthesis, whereas in *E*. *coli*, loss of deacetylase activity results in retention of immature polymer in the periplasm [6,11,17]. Studies of *Staphylococcus epidermidis* and *A*. *fumigatus* have demonstrated that the loss of exopolysaccharide deacetylation in these species results in the production of a fully acetylated glycan that is shed in the culture supernatant and does not adhere to the cell wall. This observation suggests that cationic glycans likely adhere to anionic components of the bacterial and fungal cell wall through charge.

The ability of deacetylated exopolysaccharides to adhere to microbial surfaces likely contributes to their ability to support the formation of biofilms and other microbial aggregates. Aggregate formation by *L*. *monocytogenes* and Pel-producing *P*. *aeruginosa* depends on the presence of deacetylated exopolysaccharides [6,18]. In *B*. *bronchiseptica* and likely *Klebsiella pneumoniae*, cationic glycan is necessary for the formation of the complex three-dimensional architecture of mature biofilms [2,19]. Deacetylation of exopolysaccharides also supports biofilm formation by enhancing the ability of microorganisms to adhere to host cells or abiotic surfaces. Production of deacetylated GAG and PNAG by *A*. *fumigatus*, and *S*. *epidermidis* and *Staphylococcus aureus*, respectively, has been found to enhance the adherence of these organisms to the negatively charged cell membranes of host epithelial cells. Similarly, loss of polysaccharide deacetylation impairs biofilm formation on anionic inorganic substrates, such as plastic and glass, by these organisms [1,8]. These observations strongly suggest the cationic charge of deacetylated polymers enhances adherence to anionic surfaces to support the formation of biofilms.

## Production of deacetylated exopolysaccharides mediates resistance to host defenses

Mutant organisms deficient in the production of GAG and PNAG exhibit attenuated virulence in mouse or invertebrate models of infection [1,3,7,10,19,20]. These experiments suggest that the production of cationic, adhesive exopolysaccharides also provides protection against detection and elimination by elements of the host immune system. Adhesion of exopolysaccharides to the microbial cell wall can conceal cell-surface pathogen-associated molecular patterns from immune recognition by the innate immune system. This phenomenon has been best studied in *A*. *fumigatus*, for which deletion of the deacetylase Agd3 leads to greater recognition of *β*-glucans on the surface of hyphae by pattern recognition receptor Dectin-1 [8]. Similar findings were observed with the loss of PNAG deacetylation in *S*. *epidermidis*, resulting in a mutant prone to being avidly phagocytosed by human neutrophils [1]. PNAG has also been shown to inhibit complement deposition on the cell surface of *Bordetella pertussis* [20]. The presence of Pel in *P*. *aeruginosa* biofilms provides protection from killing by the leukocyte-like cell line HL-60, although the mechanism by which this occurs has not been defined [16].

Cationic exopolysaccharides can also provide direct protection from charged antimicrobial peptides (AMPs) or other antimicrobial molecules through electrostatic interactions. Partially deacetylated PNAG of *S*. *epidermidis* enhances resistance to the microbicidal effects of the cationic LL-37 and *β*-defensin 3 AMPs, presumably through electrostatic repulsion [1]. Similar observations were made with *Aspergillus* spp., in which the production of cell wall GAG reduced the binding of neutrophil extracellular traps (NETs) to hyphae and protected the organism from NET-induced damage [13]. Deacetylation of GlcNAc residues on the cell wall surfaces of *L*. *monocytogenes* and *Streptococcus pneumoniae* renders these pathogens resistant to the positively charged lysozyme enzyme from exerting the bacteriolytic activity of cleaving bonds in the peptidoglycan layer between *N*-acetylmuramic acid and GlcNAc [21,22]. Cationic polysaccharides can also bind to and sequester anionic molecules within the biofilm matrix to prevent their access to deeper cellular structures. For example, *S*. *epidermidis* PNAG protects the integrity of biofilms from the bactericidal activity of the anionic human AMP dermcidin [23]. Deacetylated exopolysaccharides, as demonstrated in these observations, contribute to actively protecting organisms against components of the host defense.

## Deacetylated exopolysaccharides increase resistance to antimicrobials

Cationic exopolysaccharides can also mediate resistance to antimicrobial agents through repulsion or sequestration of these molecules. PNAG production increases the biofilm resistance of *Aggregatibacter actinomycetemcomitans* to the cationic detergent cetylpyridinium chloride and protects *S*. *epidermidis* against microbicidal action of glycopeptide antibiotics, such as vancomycin [9,24]. The production of GAG by *A*. *fumigatus* limits intracellular penetration of the hydrophobic antifungal posaconazole and reduces its activity [14]. Degrading Pel polysaccharide within *P*. *aeruginosa* biofilms enhanced susceptibility to colistin, and disrupting the Pel operon resulted in enhanced susceptibility of *P*. *aeruginosa* to the aminoglycosides tobramycin and gentamicin [16,18]. The ability of Pel to enhance antimicrobial resistance may be strain or condition dependent, however, because other studies have reported that disruption of the PelA deacetylase failed to alter susceptibility to tobramycin or ciprofloxacin [25]. It has been suggested that Pel polysaccharide may enhance antimicrobial resistance through interacting with and anchoring other macromolecules such as anionic extracellular DNA (eDNA) within *P*. *aeruginosa* biofilms, which in turn can act to sequester or repel antimicrobials and thereby prevent access to intracellular targets [5]. Although similar interactions of other cationic polysaccharides with eDNA have not been reported, it is likely for these cationic exopolysaccharides to have similar roles within their respective biofilms. Together, these observations suggest that the deacetylation of polymers also actively contributes to protection of their respective organisms against antimicrobials.

## Development of therapeutics that target deacetylated exopolysaccharides

Studies of the biosynthetic pathways governing the production of deacetylated exopolysaccharides have suggested that these pathogens produce hydrolytic enzymes specific for cleavage of these polymers. These enzymes include Sph3 to cleave *A*. *fumigatus* GAG, the hydrolase domain of PelA to cleave *P*. *aeruginosa* Pel, and PssZ to cleave *L*. *monocytogenes* exopolysaccharide (EPS) [6,14,26]. Cross-species activity of hydrolytic enzymes has also been demonstrated. Dispersin B (DspB) of *A*. *actinomycetemcomitans* and NghA of *Y*. *pseudotuberculosis* cleave PNAG *β*-1,6-linked GlcNAc polymers and residues, respectively, and the *P*. *aeruginosa* PelA hydrolase cleaves fungal GAG [14,27,28].

Recent studies have highlighted the potential for the use of these enzymes as therapeutic agents to target biofilm formation by their respective organisms, as well as other organisms producing polymers with shared composition and linkages. DspB has been shown to sensitize biofilms of *Actinobacillus actinomycetemcomitans* to the bactericidal effect of cationic detergent cetylpyridinium chloride, as well as anionic detergent sodium dodecyl sulfate and *Actinobacillus pleuropneumoniae* to ampicillin [24,29]. The hydrolase domain of *B*. *bronchiseptica* PgaB (PgaB_*Bb*_) from *B*. *bronchiseptica* and DspB both potentiate the killing of *S*. *epidermidis* and *E*. *coli* by gentamicin [30]. Treatment with the GAG-specific hydrolase Sph3 as well as the *P*. *aeruginosa* PelA hydrolase enhanced the antifungal activity of posaconazole, amphotericin B, and caspofungin against *A*. *fumigatus* [14]. Targeting biofilms with hydrolase enzymes also enhances the susceptibility of organisms to host defenses, because treatment of *P*. *aeruginosa* with PelA disrupted Pel-dependent biofilms and enhanced susceptibility of this organism to the antibiotic colistin as well as killing by the leukocyte-like HL-60 cell line [16]. Intratracheal treatment with recombinant Sph3 is well tolerated and attenuates virulence of *A*. *fumigatus* in an immunocompromised mouse model of invasive aspergillosis [14]. However, a recent study reported that treatment of *P*. *aeruginosa* biofilms with enzymes targeting cell wall polysaccharides resulted in dissemination of bacteria due to biofilm dispersion [31]. More work is therefore required to establish the long-term safety of these enzymes and identify the optimal organism–enzyme combinations that improve outcomes during infection.

The development of inhibitors of exopolysaccharide deacetylases is another promising therapeutic strategy. Although this work remains in its infancy, several metal chelating inhibitors have been synthesized to inhibit *E*.*coli* PgaB (PgaB_*Ec*_) deacetylation of PNAG [32–34].

## Future directions

Although our understanding of the biosynthetic pathways governing the synthesis of deacetylated exopolysaccharides has expanded greatly, there are many unanswered questions about their functions in host–pathogen interactions. For example, studies of *A*. *fumigatus* GAG suggest that purified fractions of this glycan can directly modulate host immune responses, although the receptors and signaling pathways governing this process remain unknown [35,36]. These and similar studies in other organisms await robust protocols for the purification of deacetylated exopolysaccharides or the production of synthetic oligosaccharides derived from these glycans. In addition, the function of deacetylated exopolysaccharides has been largely studied in the context of single-species biofilms. However, as these organisms typically exist in polymicrobial environments, the potential for exopolysaccharides to play cooperative roles in multispecies biofilms also needs to be explored.
